# Phenotyping breast cancer cell lines EM-G3, HCC1937, MCF7 and MDA-MB-231 using 2-D electrophoresis and affinity chromatography for glutathione-binding proteins

**DOI:** 10.1186/1471-2407-10-449

**Published:** 2010-08-23

**Authors:** Jana Mladkova, Miloslav Sanda, Eva Matouskova, Irena Selicharova

**Affiliations:** 1 Institute of Organic Chemistry and Biochemistry, Academy of Sciences of the Czech Republic v.v.i., Prague, Czech Republic; 2 Institute of Biochemistry and Experimental Oncology, 1st Faculty of Medicine, Charles University, Prague, Czech Republic; 3 Prague Burn Centre, 3rd Faculty of Medicine, Charles University, Prague, Czech Republic

## Abstract

**Background:**

Transformed phenotypes are common to cell lines derived from various cancers. Proteome profiling is a valuable tool that may reveal uncharacteristic cell phenotypes in transformed cells. Changes in expression of glutathione S-transferases (GSTs) and other proteins interacting with glutathione (GSH) in model cell lines could be of particular interest.

**Methods:**

We compared the phenotypes of breast cell lines EM-G3, HCC1937, MCF7 and MDA-MB-231 using 2-D electrophoresis (2-DE). We further separated GSH-binding proteins from the cell lines using affinity chromatography with GSH-Sepharose 4B, performed 2-DE analysis and identified the main protein spots.

**Results:**

Correlation coefficients among 2-DE gels from the cell lines were lower than 0.65, pointing to dissimilarity among the cell lines. Differences in primary constituents of the cytoskeleton were shown by the 2-D protein maps and western blots. The spot patterns in gels of GSH-binding fractions from primary carcinoma-derived cell lines HCC1937 and EM-G3 were similar to each other, and they differed from the spot patterns of cell lines MCF7 and MDA-MB-231 that were derived from pleural effusions of metastatic mammary carcinoma patients. Major differences in the expression of GST P1-1 and carbonyl reductase [NADPH] 1 were observed among the cell lines, indicating differential abilities of the cell lines to metabolize xenobiotics.

**Conclusions:**

Our results confirmed the applicability of targeted affinity chromatography to proteome profiling and allowed us to characterize the phenotypes of four breast cancer cell lines.

## Background

Cell lines provide indispensable tools in many aspects of laboratory research, particularly as *in vitro *models for cancer research. About 6,500 new articles related to cancer are retrieved every 30 days in PubMed, and nearly 2,000 articles are retrieved with the key word 'cell line'.

Despite these advantages, the use of cell lines in cancer research is also disputatious [1]. A common argument for this is that cell lines are not representative of the actual tumor diversity and heterogeneity [2].

It should be stressed that the use of different cancer cell lines without considering their unique phenotypes is a common problem neglected by many investigators. For some *in vitro *applications (such as toxicological assessments), it is essential to know the exact differentiation state and functionality of the cells being used. This can be approached through proteomic profiling [3-5]. However, analysis of the complete proteome is a complicated task. A common method for proteome analysis combines the protein separation by two-dimensional electrophoresis (2-DE) with mass spectrometric (MS) identification of selected protein spots [6,7]. Although 2-DE is capable of analyzing several thousand proteins simultaneously, there are significant limitations to this method. 2-DE cannot detect some potentially important proteins such as hydrophobic membrane proteins and proteins with higher relative molecular mass. Another problem is detection of proteins over their natural range of abundance [8].

A combination of affinity chromatography with proteomic methodology is an attractive approach that may lead to selective pre-fractionation of groups of proteins of interest and enable detection of less abundant proteins [9,10]. The expression of glutathione S-transferases (GSTs) and other proteins interacting with glutathione (GSH) in model cell lines is of particular interest. GSTs are a superfamily of detoxification enzymes, and GSH is in the front line of cellular defense against oxidative stress [11,12]. Levels of GST expression are potentially important determinants in the susceptibility of tissues to the mutagenic effects of chemical carcinogens and the response of tumors to chemotherapy [11]. The impact of GST genetic polymorphisms on human cancer susceptibility has been addressed by many studies [13]. GSTs are considered protein markers for individuation of chemotherapy [14,15]. Inhibitors of GSTs can potentially be utilized to overcome tumor cell drug resistance, or pro-drugs can be designed that are activated by GSTs and thereby take advantage of its overexpression [16].

The proportion of proteins interacting with GSH and enzymes metabolizing GSH in a cell can potentially denote the ability of a cell to survive in unfavorable conditions [12]. In this study, we set out to enrich for GSH-binding proteins from model breast cancer cell lines.

The most widely used breast cancer cell lines are MCF7, established in 1973 [17], and MDA-MB-231 established in 1974 [18]. Both are derived from pleural effusions of metastatic mammary carcinoma patients. Line MCF7 expresses markers of the luminal epithelial phenotype of breast cells and is used as a model for estrogen receptor-positive tumors [19]. The MDA-MB-231 line does not express epithelial markers but contains a high level of vimentin, a marker of the mesenchymal phenotype. It is used as a model for estrogen receptor-negative and HER-2/neu-negative breast cancers [20]. More recently, several permanent cell lines were derived from primary breast tumors. Among these, the line HCC1937 was isolated in 1998 from a primary carcinoma of a germ-line BRCA1 mutation carrier [21]. Cell line EM-G3, derived from a primary lesion of infiltrating ductal breast carcinoma, was established in 2006 [22]. It expresses mixed markers for the basal and luminal phenotypes and exhibits differentiation potential.

We separated the GSH-binding proteins in these cell lines using affinity chromatography on GSH-Sepharose 4B, performed 2-DE analysis of proteins with affinity to GSH and identified the main protein spots.

We also compared phenotypes of the breast cell lines MCF7, MDA-MB-231, HCC1937 and EM-G3 using 2-DE profiling. We have previously published the 2-DE map of the EM-G3 cell line [23]. We prepared 2-DE maps of the MCF7, MDA-MB-231 and HCC1937 cell lines and analyzed the positions and intensities of the main cellular protein spots. 2-DE is shown to be an easy tool for straightforward comparison of cell phenotypes.

Besides compelling phenotypic differences, we found considerable differences in the content of GSTs and other GSH-binding proteins among the selected cell lines.

## Methods

### Cell culture and sample preparation

The human breast cell line EM-G3 and primary cultures of normal mammary epithelial (NME) cells from four donors (NME23, NME35, NME36 and NME37) were grown as described [22,24]. The samples were obtained from women who underwent breast reduction at the General Faculty Hospital in Prague. The written patient's informed consent approved by the Ethical committee of the General Faculty Hospital in Prague was obtained prior to surgery.

Human breast cancer cell lines MDA-MB-231, MCF7 and HCC1937 were obtained from American Type Culture Collection (ATCC). Cells were maintained in MEM supplemented with 10% bovine serum, 2% fetal bovine serum, 2 mM L-glutamine, 100 U/ml penicillin and 100 μg/ml streptomycin.

Cells grown to confluence were washed with PBS then harvested by treatment with trypsin/EDTA in PBS. Cells were pelleted by centrifugation at 800 g for 10 min. Cell pellets were washed three times with PBS and stored in aliquots of 5 × 10^6 ^cells at -70°C.

Cell pellets destined for 2-DE analysis were lysed for 1 h in denaturing lysis buffer composed of 7 M urea, 2 M thiourea, 4% (w/v) CHAPS, 40 mM Tris, 65 mM DTT and 2% (v/v) ampholytes (pH 9-11), using 60 μl per 10^6 ^cells. The lysate was centrifuged at 16,000 g for 10 min at 20°C.

Cell pellets destined for affinity chromatography and/or enzyme activity measurements were lysed for 30 min in non-denaturing lysis buffer using 60 μl per 10^6 ^cells. The buffer was composed of 20 mM Tris-Cl pH 8.0, 0.1% (w/v) CHAPS, 0.1 M NaCl, 0.25 M sucrose, 5 mM DTT and 1 μl per 10^6 ^cells of protease inhibitor cocktail P8340 (Sigma, USA). Cells were homogenized for 30 min at 0°C and sonicated three times for 2 s. The lysate was centrifuged at 9,000 g for 10 min at 4°C, resuspended (using 10 μl buffer per 10^6 ^cells), and the combined supernatants were centrifuged at 16,000 g for 20 min at 4°C.

Protein content was determined using the Bradford assay [25]. Samples were stored in aliquots at -70°C.

### Affinity chromatography on GSH-Sepharose 4B

The lysates from 10^7 ^cells of each cell line (EM-G3, MCF7, MDA-MB-231 and HCC1937) in non-denaturing buffer were applied to GSH-Sepharose 4B (GE Healthcare, Sweden) columns (1 × 1.5 cm). Affinity chromatography was performed according to the manufacturer's protocol with small modifications concerning buffer supplementation and increased concentration of GSH in elution buffer. All procedures were performed at 4°C. Briefly, samples were incubated for 1 hour with resin then washed with PBS supplemented with 0.05% (v/v) protease inhibitor cocktail and 5 mM DTT. The adsorbed proteins were eluted with 10 mM Tris-Cl pH 8.0 containing 5 mM GSH and 0.05% (v/v) protease inhibitor cocktail.

The course of affinity chromatography was monitored by absorbance at 280 nm and GST activity measurements. Flow through fractions and GSH-eluted fractions were concentrated and desalted using a Microcon centrifugal filter device (Millipore, USA) with a 10 kDa exclusion limit. GST activity was determined in the samples. The samples destined for 2-DE were then mixed with IEF rehydration buffer (7 M urea, 2 M thiourea, 4% (w/v) CHAPS, 50 mM DTT and 0.8% (v/v) ampholytes (pH 3-10)).

### Glutathione S-transferase activity

GST activity was measured according to the method of Habig et al. [26] using 1-chloro-2,4-dinitrobenzene (CDNB) as the substrate. The reaction mixture (2 ml) contained 50 mM potassium phosphate buffer pH 6.5, 1 mM GSH and 1 mM CDNB. The mixture was incubated for 10 min at 25°C and the absorbance change at 340 nm was measured using a Lambda 25 UV/VIS spectrophotometer (Perkin Elmer, USA). Each determination was performed in triplicate. Specific GST activity was calculated using the *S*-(2,4-dinitrophenyl) glutathione extinction coefficient ε_340 _= 9.6 mM^-1^·cm^-1^. One unit of enzymatic activity is defined as the amount of enzyme that catalyzes the formation of 1 μmol product per minute under the specified conditions. Specific activity was expressed as mM product per minute per mg protein.

### 2-DE

The cell lysates in denaturing buffer (70 μg or 15 μg of protein) were applied to 18-cm linear IPG strips pH 4-7 or to 7-cm IPG strips pH 3-10 (GE Healthcare, Sweden).

The desalted and concentrated fractions after affinity chromatography on GSH-Sepharose 4B that had been transferred to IEF rehydration buffer were applied to 7-cm linear IPG strips pH 3-10.

2-DE was performed as described before [23], except 12% (4% stacking gel, 19 × 22 cm or 8 × 8 cm) polyacrylamide gels were used instead of gradient gels. The analytical gels were stained with silver stain according to the Bloom method as modified by Rabilloud [27].

Preparative 1-D and 2-D gels (18-cm IPG strips pH 3-10) were prepared from the fractions eluted by GSH from the GSH-Sepharose 4B. Preparative gels were stained with colloidal Coomassie [28].

### Image analysis

Gels were scanned with a GS-800 Calibrated Densitometer (Bio-Rad, Hercules, CA) at 400 dpi resolution. Gels were analyzed using PDQuest Advanced 8.0.1 2D Gel Analysis Software (Bio-Rad, Hercules, CA). The locations and intensities of spots in gels from whole cell lysates differed considerably. The gels were analyzed in pairs to minimize any mismatching of spots that was pertinent to complex match sets. The same detection parameters were used for each analysis. Correlation coefficients among cell lines were determined after manual editing of spot matching. Analysis of technical replicates of gels from each cell line under the same conditions was performed.

A match set was created from gels of GSH-binding fractions. Spot detection and matching were edited manually. Correlation coefficients among gels were determined. Spot intensities in 2-DE gels of GSH-binding proteins eluted from the affinity columns were estimated after normalization to the total density of the image.

### Mass spectrometry and protein identification

Proteins were excised from preparative gels and in-gel digested with trypsin. Extracted peptides were concentrated in a SpeedVac (Thermo Fisher Scientific, USA). The resulting peptide mixture was analyzed by LC-MS/MS on an LTQ-ORBITRAP mass spectrometer (Thermo Fisher Scientific, Germany) coupled to a Rheos 2000 2-D capillary chromatograph (Flux Instruments, Switzerland). The first dimension column was a Symetry C18, 180 μm × 20 mm × 3 μm (Waters, UK) and the second dimension column was a PepMap C18, 75 μm × 150 mm × 3 μm (LC Packings, USA). A data-dependent scan composed of one full MS scan (resolution of 60,000) and three CID MS/MS scans (resolution of 7,500) was used as the mass spectrometry method. The mass tolerance for peptide identification was 10 ppm and for searching fragment ions was 50 ppm. The identities of all peptides were confirmed by at least three fragment ions. Sequence searches were performed against the Uniprot protein database (version accessed at November 1, 2008) by Bioworks Browser 3.3.1 SP1 and Sequest 2.0 software (Thermo Fisher Scientific, USA).

### Immunoblotting

Cell lysates (20 μg protein) in denaturing buffer were separated by SDS electrophoresis (12% SDS polyacrylamide gels) and electroblotted onto nitrocellulose membranes at 12 V for 15 min in 20 mM CAPS/NaOH buffer pH 10.3 containing 10% (v/v) methanol and 0.1% (w/v) SDS. All incubations and washes were performed at room temperature with 50 mM Tris/HCl buffer pH 7.5 containing 140 mM NaCl and 0.1% (v/v) Tween 20 (T-TBS). Membranes were blocked with 3% (w/v) bovine serum albumin in T-TBS overnight. Membranes were then incubated for 1 h with rabbit anti-actin antibody (A5060, Sigma, USA), mouse monoclonal anti-vimentin antibody (RTU-VIM-V9, Novocastra, UK), mouse monoclonal anti-CK19 antibody (NCL-CK19, Novocastra, UK) and mouse monoclonal anti-CK13 antibody (NCL-CK13, Novocastra, UK).

Membranes were washed with T-TBS and incubated for 1 h with anti-rabbit peroxidase-conjugated antibody (A9169, Sigma, USA) or anti-mouse peroxidase-conjugated antibody (A9044, Sigma, USA). The membranes were then washed with T-TBS and developed with diaminobenzidine or SuperSignal West Pico Chemiluminescent substrate (Pierce, Rockford, IL) and detected with a CCD camera Las-3000 (Fujifilm Life Science, USA).

## Results

### Phenotypes of model cell lines

We prepared 2-DE protein maps for the MCF7, MDA-MB-231 and HCC1937 cell lines in the pH range 4-7 and compared them with our 2-DE protein map of the EM-G3 cell line published in 2007 [23]. We performed image analysis of the cell lines using PDQuest software. Although the main cellular proteins could be matched easily among the cell lines, the 2-DE protein maps differed considerably, pointing to the already known phenotypic differences among the lines. Quantitative comparisons or searches for differentially expressed spots have not been performed. Correlation coefficients among 2-D gels were used as a measure of similarity. Cell lines were compared to each other, with a single image of each cell line used to estimate the number of spots and correlation coefficients among the lines. The correlation coefficients between two technical replicates of gels from each cell line were also determined. In total 10 analyses were performed. The correlation coefficients are shown in Table 1. The correlation coefficients among lines were lower than 0.65, indicating their dissimilarity, while correlation coefficients between technical replicates exceeded a value of 0.80, indicating that high reproducibility was achieved with our methodology [29].

**Table 1:** Correlation coefficients among cell lines and number of spots detected in each cell line

	EMG3	HCC1937	MDA-MB231	MCF7	spots^a^	cor. coef.^b^
EMG3	-	0.63	0.55	0.62	361 ± 13	0.88
HCC1937		-	0.62	0.65	388 ± 19	0.91
MDA-MB231			-	0.46	401 ± 9	0.81
MCF7				-	344 ± 21	0.80

a) Number of spots detected in the gels from four analyses.

b) Correlation coefficient between two technical replicates of gels from a given cell line.

**Figure 1:**
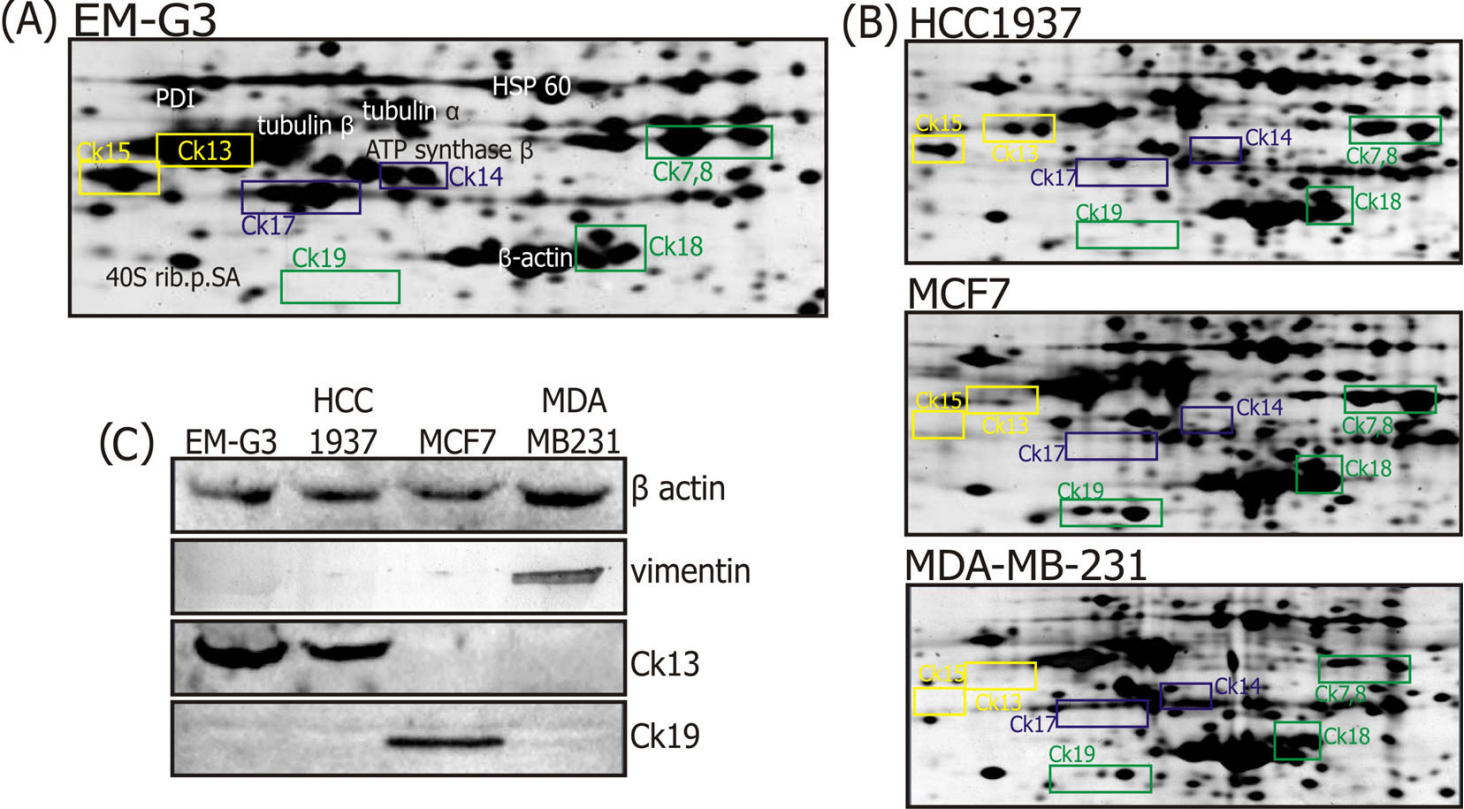
**Main constituents of cytoskeletons from the breast cell lines**. Sections of the 2-DE gels (Mr 38-60, pI 4.6-5.7) from breast cell lines showing the main constituents of the cytoskeleton. (A) The identities of spots are shown for cell line EM-G3. Spots corresponding to CKs are framed. Simple CKs are in green, basal CKs are in blue and CKs atypical for mammary glands are in yellow. The data were subtracted from Selicharova et al. [23]. Protein names are abbreviated as 40S rib.p.SA = 40S ribosomal protein SA, CK = cytokeratin, HSP = heat shock protein and PDI = protein disulfide isomerase. (B) Estimated positions of CKs are shown for the cell lines HCC1937, MCF7 and MDA-MB-231. (C) Immunoblot analysis. Proteins from each cell lysate were separated by 1-D SDS-PAGE and detected with rabbit anti-actin antibody and mouse monoclonal anti-vimentin, anti-CK19 and anti-CK13 antibodies.

The main cytoskeletal constituents of individual cell lines are shown in Figure 1, together with Western blots showing expression of actin, vimentin, CK19 and CK13. High levels of mixed (basal and luminal) cytokeratins were characteristic for the cell line EM-G3 [23]. The MCF7 cells are known to express only the simple epithelial CKs characteristic of the luminal mammary cell phenotype [30]. MDA-MB-231 cells contain small amounts of CKs but express the mesenchymal marker vimentin [30]. The HCC1937 cell line does not express basal CKs, and might be expected to express simple CKs that are typical for luminal cells, as suggested from our 2-DE protein maps. However, it does not express CK19 but surprisingly contains a discernible amount of CK13. Expression of CK13, which is not considered a typical mammary gland cytokeratin, was observed in the EM-G3 cell line [23].

### GST activity in breast cell lines and primary cultures of NME cells

**Figure 2:**
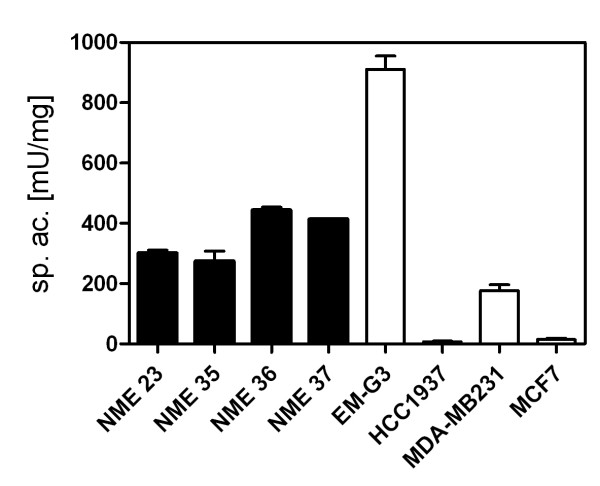
**Specific activities of GST**. Specific activities of GST in four primary cultures (black columns) of NME cells (NME23, NME35, MNE36 and NME37) and in permanent breast cell lines (white columns).

The GST activity was measured in lysates from cells in non-denaturing buffer. GST activities in lysates from the permanent cell lines and four primary cultures of NME cells were determined. The data are presented in Figure 2. The GST activities in the primary cultures ranged between 200-500 mU/mg cell protein. Major differences in GST activity were observed among the permanent cell lines. The activity in MDA-MB-231 was about 200 mU/mg, the activity in EM-G3 was the highest (912 mU/mg) while the GST activities in the lines MCF7 and HCC1937 were negligible.

### 2-DE analysis of protein fractions retained on GSH-Sepharose

Proteins fractions from lines EM-G3, HCC1937, MCF7 and MDA-MB-231 were eluted from GSH-Sepharose columns with 5 mM GSH, concentrated and desalted by ultrafiltration. The GST specific activity of eluted fractions was about 100-200 fold higher then in cell lysates, but the yields for the activity were only about 10-30% of the total in the lysates before affinity chromatography. The protein concentrations in the eluted fractions were very low.

**Figure 3:**
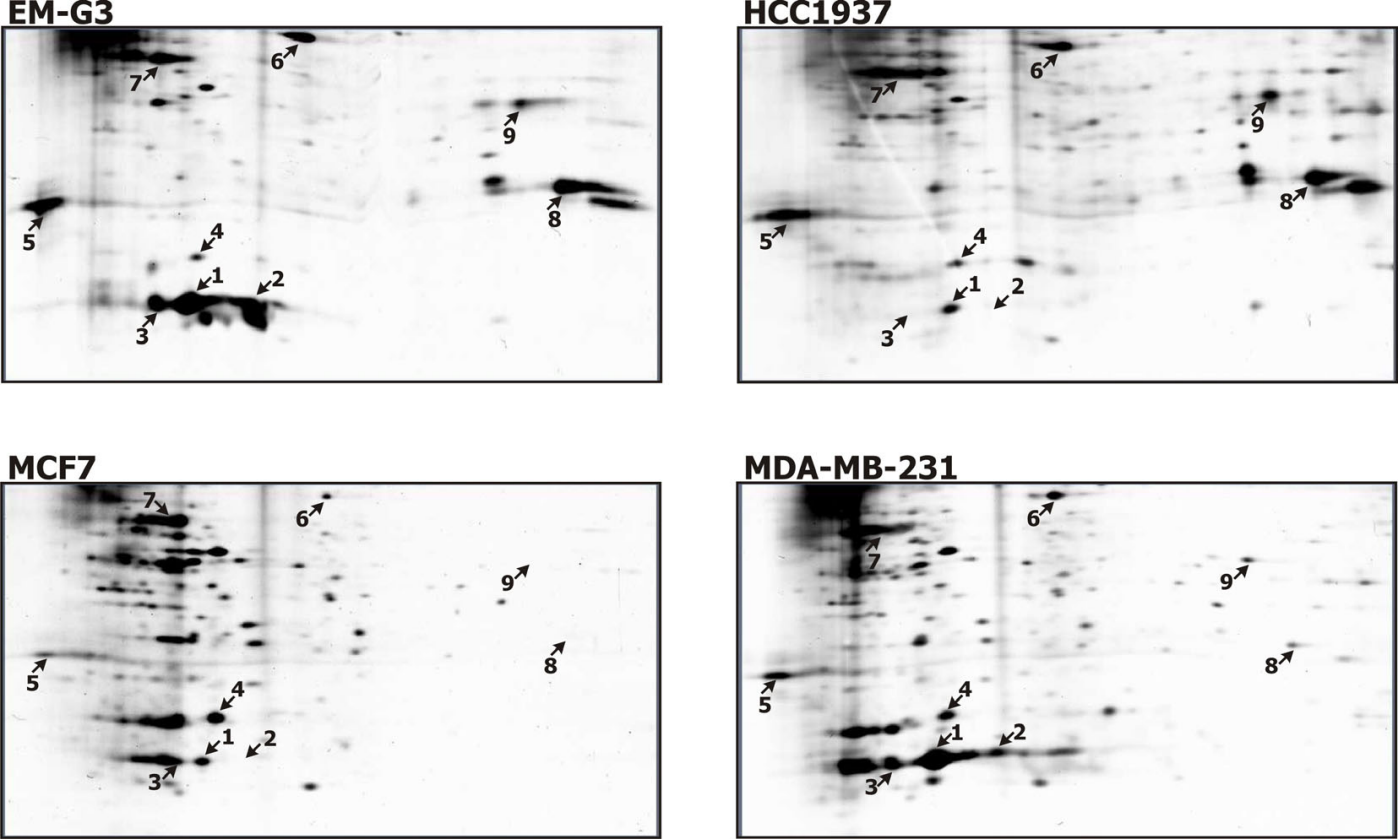
**GSH-binding proteins from the breast cell lines**. Sections of the 2-DE gels (Mr 20-52, pI 4.3-9.3) for GSH-binding proteins from EM-G3, HCC1937, MCF7 and MDA-MB-231 cell lines. Desalted and concentrated fractions from the affinity chromatography were focused on 7 cm IPG strips pH 3-10 and separated in 12% SDS-PAGE gels. The proteins identified with numbered spots are listed in Table 3.

2-DE gels of fractions from cell lines eluted with 5 mM GSH from GSH-Sepharose 4B columns are shown in Figure 3. The 2-DE protein maps of GSH-binding proteins contained several intensive spots and many faint spots. The maps differed considerably among cell lines. The main features distinguishing the protein maps were the intense spots numbered 1 and 8 (Figure 3). We performed image analysis of the gels using the PDQuest software. A single image of each GSH-binding fraction was used to estimate the number of spots and correlation coefficients among the gels. The correlation coefficients are shown in Table 2. Although the activity of GST was negligible in lines HCC1937 and MCF7 as compared to lines EM-G3 and MDA-MB-231, the overall spot patterns showed closer similarity between lines HCC1937 and EM-G3, derived from the primary tumors, when compared to the MDA-MB-231 and MCF7 lines derived from pleural effusions of metastatic breast cancers.

**Table 2:** Correlation coefficients among the GSH-binding fractions and number of spots detected in each cell line

	EMG3	HCC1937	MDA-MB231	MCF7	spots
EMG3	-	0.80	0.65	0.64	127
HCC1937		-	0.55	0.54	137
MDA-MB231			-	0.68	154
MCF7				-	132

### Protein identification

The fractions eluted by 5 mM GSH from GSH-Sepharose 4B contained very small amounts of protein. Only major spots were visible in preparative gels. We succeeded in identifying nine spots (Table 3). Seven spots were identified from 2-DE gels, and two more proteins were identified from bands in 1-D gels and their positions in 2-DE gels were estimated (Figure 3). The relative quantities of the spots in each cell line, as determined from the PDQuest analysis, are presented in Table 4. The values were normalized to total density in the gel image and are expressed as thousandths of the total density.

**Table 3:** Proteins identified from GSH-binding fractions of breast cell lines

Spot no.	Accession no.	Protein name	Theor. Mr/pI	Meas. Mr/pI	Seq. cov. (%)	Pept. seq.
1	P09211	GST P1-1	23,2/5,4	25/5,5	52	15
2	P09211	GST P1-1	23,2/5,4	25/5,7	47	7
3	P09211	GST P1-1	23,2/5,4	25/5,3	51	10
4	P21266	GST M3-3	26,4/5,4	27/5,6	33	8
5	P24534	EF 1-β	24,6/4,5	30/4,5	24	6
6	P26641	EF 1-γ	50,0/6,3	50/nd^a^	18	7
7	P60709	β-actin	41,6/5,3	44/5,3	18	7
8	P16152	Carbonyl reductase [NADPH] 1	30,2/8,5	32/8,5	20	5
9	O43813	LanCL 1	45,3/7,9	40/nd^a^	5	2

For each protein spot we show the accession number in the Swiss-Prot database, the protein name or its abbreviation, the theoretical (Theor.) and experimental (Meas.) relative molecular weight (in kDa) and isoelectric point (Mr/pI), the sequence coverage and the number of peptides sequenced. Protein names are abbreviated as GST = glutathione S-transferase, EF = elongation factor, LanCL 1 = LanC-like protein 1.

a) The proteins were identified from 1-D SDS-PAGE gels, and their positions in 2-D gels were estimated according to their Mr/pI (Figure 3).

**Table 4:** Relative intensities of spots in the gels from GST-binding fractions

Spot no.	Accession no.	Protein name	Relative intensity of spots^a^			
			EM-G3	HCC1937	MDAMB231	MCF7
1	P09211	GST P1-1	49	6	48	6
2	P09211	GST P1-1	16	-	7	-
3	P09211	GST P1-1	10	-	11	3^b^
4	P21266	GST M3-3	2	3	6	12
5	P24534	EF 1-β	11	26	5	2
6	P26641	EF 1-γ	8	8	3	1
7	P60709	β-actin	17	34	24	32
8	P16152	Carbonyl reductase [NADPH] 1	15	22	1	-
9	O43813	LanCL 1	4	9	14	-

For each protein spot we show the accession number in the Swiss-Prot database, the protein name or its abbreviation. Relative intensities of spots in the gels were estimated by PDQuest analysis. Protein names are abbreviated as GST = glutathione S-transferase, EF = elongation factor, LanCL 1 = LanC-like protein 1.

a) The values were normalized to total density in the gel image and are expressed as thousandths of total density.

b) The measured density corresponds to a proportional part of a diffuse spot that overlaps the respective position of spot no.3 (Figure 3).

The predominant protein retained on the GSH-affinity column was GST P1-1, identified in three spots (no. 1, 2 and 3). The differences in pIs of the detected GST P1-1 isoforms might be caused by post-translational modifications or they might rose from polymorphism of the enzyme [Swiss-Prot:P09211]. These spots were dominant in the EM-G3 and MDA-MB-231 cell lines and almost undetectable in the MCF7 and HCC1937 cell lines. This is in accordance with the GST activity measurements. We further identified GST M3-3 (spot no. 4). This spot had a comparable intensity in each cell line. Another large spot (no. 8) was identified as carbonyl reductase [NADPH] 1. This protein was present in the EM-G3 and HCC1937 cell lines and missing in the MCF7 and MDA-MB-231 cell lines. We further identified elongation factors EF-1β (spot no. 5) and EF-1γ (spot no. 6) together with β-actin (spot no. 7) and lanC-like protein 1 (LanCL 1, spot no.9).

## Discussion

Our study had two goals: 1) to perform a basic comparison of breast cell line phenotypes by comparing protein 2-DE maps; and 2) to analyze the cellular sub-proteome interacting with GSH in breast cell lines. Our results confirmed the applicability of targeted affinity chromatography to proteome profiling and allowed us to characterize the phenotypes of four breast cell lines.

Proteome profiling is a valuable tool providing information about the phenotype, functionality and differentiation state of the cells. The challenge is that a large number of proteins need to be clearly related to specific functions. Suitable reference and control samples should be included in the analysis. This advanced methodology for proteomic analysis and protein identification should be combined with extensive bioinformatic searches [4,5].

Here, we present a simplified approach that can be utilized for basic comparisons of phenotypes for selected cell lines. Major differences in proteomes of cell lines selected for any application can be recognized directly in a single 2-DE gel. Based on their similarity or dissimilarity a decision about usage of the cells can be made. Analysis of whole cell lysates or of samples from targeted pre-fractionation might also be used.

In this work, we took advantage of the previously characterized proteome from the cell line EM-G3 [23]. Correlation coefficients among 2-D gels were used as a measure of similarity [29]. Although all cell lines originated from breast cancer, their phenotypes were completely different as evidenced from our analysis. When comparing whole cell lysates, the most diverse cell lines were MCF7 and MDA-MB-231. However, this was not the case when we compared the GSH-binding fractions. The spot patterns in gels from cell lines HCC1937 and EM-G3 derived from primary carcinomas were mutually more similar, and differed from the spot patterns in the cell lines MCF7 and MDA-MB-231 that are derived from pleural effusions of metastatic mammary carcinoma patients. Extensive changes in the GSH-binding sub-proteome may be a characteristic feature of the metastatic cell phenotype [14,31]. Alternatively, the changes in GSH-binding proteomes might be partially caused by extensive cultivation of the MCF7 and MDA-MB-231 cell lines that were established long time ago (in 1971 respectively in 1974) compared to the relatively "younger" cell lines HCC1937 and EM-G3.

The GST activities among breast cell lines differed widely. An inverse correlation between estrogen receptor (ER) status and GST pi expression has been reported for breast cancer [32]. This might explain the decreased levels of GST pi in the MCF7 cell line, which is ER positive, and increased levels in the EM-G3 and MDA-MB-231 cell lines that are ER negative. However it does not explain the low levels of GST activity in the HCC1937 cell line, which is also ER negative. Variability in localization of GST pi between normal epithelial cells and tumor cells was reported by Forrester et al. [33]; further, in some of their samples, GST pi was almost absent from tumor tissue. Buser et al. [14] suggested that low levels of GST activity are associated with more aggressive and more advanced disease, while high levels of GSTs are part of the initial phenotype of malignant cells. We detected the highest specific activity of GST in the cell line EM-G3, which represents a pre-malignant population of mammary epithelial cells progenitors [22]. It is clear that expression of GST varies among breast cancers. Exact determination of its expression is crucial to ascertain the utility of drugs based on GST inhibitors or activated by GST [16].

Comparison of 2-DE protein maps for proteins eluted from GSH-Sepharose confirmed differences in the measured specific activities of GSTs and revealed deeper divergences among cell lines.

We succeeded in identifying only the most abundant proteins retained on the column. Faint spots were undetectable in the preparative gels. The main proteins identified were GST P1-1 in three spots, GST M3-3 and carbonyl reductase [NADPH] 1. All of these proteins are involved in the metabolism of xenobiotics. GSTs catalyze conjugation of glutathione with a wide variety of electrophilic compounds [11]. Carbonyl reductase [NADPH] 1 catalyzes reduction of a wide variety of carbonyl compounds including quinones, prostaglandins and various xenobiotics [34]. It has high affinity for GSH-conjugated substrates. Major divergences in expression for these enzymes among the cell lines were observed. Carbonyl reductase was missing in lines MCF7 and MDA-MB-231, which are of metastatic origin. GST P1-1 was largely decreased in lines MCF7 and HCC1937. These cell lines showed some similarity when analyzing whole cell lysates, probably reflecting their relation to the luminal phenotype of mammary cells as based on their CK expression profiles [30].

Surprisingly we have not identified other typical GSH-binding proteins, such as GSH peroxidase, GSH reductase or glyoxalase. These proteins were probably retained on the column but not concentrated in sufficient quantities for identification.

We also detected β-actin, which is one of the most abundant proteins in cell lysates and was likely retained on the column because it is a "sticky" protein that interacts with many partners. Actin polymerization is regulated by reversible glutathionylation, thus the interaction with glutathione might be specific to a certain extent [35].

We also identified two subunits of elongation factor 1 (EF-1): EF1-β and EF1-γ. EF1-β contains a GST C-terminal domain and, together with EF1-δ, stimulates the exchange of GDP that is bound to EF-1-alpha for GTP. EF1-γ contains both GST C-terminal and N-terminal domains and probably plays a role in anchoring its complex to other cellular components [36]. The role of GST domains in elongation factor subunits is not clear. They might be involved in the regulation of protein folding and assembly [37].

The final protein identified was LanCL1. LanCL1 is a mammalian ortholog of the prokaryotic enzyme lanthionin synthase and its function in eukaryotes is unknown [38]. Similar to our analysis, LanCL1 is one of the main proteins from bovine brain lysates retained on GSH-Sepharose [39]. It has been suggested that LanCL1 might be significant for neurodegenerative diseases and in cellular signaling and differentiation [38].

## Conclusions

To conclude, we analyzed the four breast cell lines EM-G3, HCC1937, MCF7 and MDA-MB-231, and showed major differences in their phenotypes especially in GSTs activities and GSH-binding proteomes. It can be deduced that such diversity might exist among other cell lines and among activities of many other proteins. Various cell lines are often used in parallel. They are employed in determinations of bioactivities for various compounds [40,41], in cytotoxicity measurements [42,43] and also in studies of specific protein functions and genes after gene manipulations [44,45]. Such comparisons of the behavior of cell lines should be interpreted considering their phenotypes. Particularly, relevance of GST activity for toxicological studies is obvious. Expression of GSTs in cell lines selected as models for toxicological studies should be determined and possibility of metabolization of tested compounds should be considered.

The usefulness of targeted affinity in proteome profiling has been demonstrated. However, when applying this methodology certain issues should be taken into account. The main restriction is a significant consumption of a sample, while the yields of proteins retained on affinity columns are very low. Introducing numerous steps to sample handling is a source of contamination and undefined protein losses. Affinity chromatography is not a scavenger method, and complicated affinity equilibria for various proteins towards the ligand should be anticipated [46]. Nevertheless, our study produced compelling data that validates our approach.

## List of abbreviations

2-DE: two dimensional electrophoresis; CK: cytokeratin; GSH: glutathione; GST: glutathione S-transferase; MS: mass spectrometry; NME: normal mammary epithelial

## Competing interests

The authors declare that they have no competing interests.

## Authors' contributions

**JM **performed affinity chromatography, GST activity measurements and prepared 2-DE gels, prepared the samples for identification of proteins and contributed to writing of the manuscript. **MS **performed the LC-MS/MS experiments and protein identifications. **EM **established and handled the cell cultures. **IS **designed and coordinated the study, performed the analysis of 2-DE gels and Western blots and drafted the manuscript. All authors read and approved the final manuscript.

## Pre-publication history

The pre-publication history for this paper can be accessed here:

http://www.biomedcentral.com/1471-2407/10/449/prepub
